# Weight loss and lifestyle change among high-risk individuals enrolled in a digital diabetes prevention program: A longitudinal study of private and public health insurance members in Western New York

**DOI:** 10.1016/j.pmedr.2023.102507

**Published:** 2023-11-10

**Authors:** Sarah LaPointe, Michael Merrill

**Affiliations:** a Department of Epidemiology, Rollins School of Public Health, Emory University, GA, USA; b Research Department, Brook, Inc., Seattle, WA, USA

**Keywords:** Diabetes, Weight loss, DPP, Medicare, Prediabetes

## Abstract

Weight loss is critical to reduce diabetes risk. Diabetes Prevention Programs (DPPs) are effective for weight loss, although less is known about digital DPPs. This study explores the association between Brook+, a 52-week digital DPP, and weight loss. This longitudinal observational study uses a sample of 5,516 private, Medicare, and Medicaid health insurance members from Western New York enrolled into Brook+ between December 2020 and December 2022. We used multivariable generalized linear regression models to estimate the association between completion of the Brook+ program, overall and stratified by health insurance type, and 5% weight loss using odds ratios (OR) and 95% confidence intervals (CI), adjusted for age and gender. We also estimated average weight loss from baseline associated with high engagement with the program using adjusted linear regression models. In the pooled sample, those who completed the Brook + program had 21% increased odds of 5% weight loss (OR = 1.21 95% CI: (1.02 – 1.44)). Among users enrolled in private, Medicare, and Medicaid health insurance, program completion was associated with 21%, 33%, and 13% increased odds of 5% weight loss, compared to those who did not complete Brook+. Interacting with health coaches, increased physical activity, and meal logging were all significant predictors of weight loss. Our results suggest that digital DPPs are promising for large-scale diabetes prevention via weight loss and lifestyle change.

## 1 Introduction

Diabetes is a prevalent and costly disease that affects 8 % of the United States (US) population (CDC, 2023). It is a leading cause of death in the US and costs associated with diabetes were estimated to be $327 billion as of 2017 ([5], CDC, 2023). There are a range of comorbidities that accompany diabetes, including hypertension, obesity, polycystic ovarian syndrome, dyslipidemia, obstructive sleep apnea, and nonalcoholic fatty liver disease (Teck, 2022). Type 2 diabetes accounts for more than 90 % of all diagnosed cases of diabetes (CDC, 2022). Type 2 diabetes follows a trajectory of elevated blood glucose concentrations referred to as prediabetes and individuals in this stage represent a high-risk group to target for prevention strategies.

Evidence suggests that effective treatment and prevention strategies for diabetes and comorbid conditions include medication and lifestyle interventions. A landmark study conducted by the Diabetes Prevention Program Research Group (DPPRG) found that an intensive lifestyle intervention led to greater reductions in diabetes incidence compared to metformin or placebo (Knowler, 2002). A long-term (15-year follow-up) study conducted by the DPPRG among the same sample found sustained reductions in incidence of diabetes, which were greatest among the lifestyle intervention group, compared to placebo (Diabetes Prevention Program Research Group, 2015). The results of these two studies in concert with a growing body of literature suggest that lifestyle interventions including dietary changes and increased physical activity targeting weight reduction are key to the prevention and management of diabetes (Gruss, 2019). More specifically, among these modifiable risk factors for diabetes, weight loss has been shown to be the most effective approach to diabetes prevention (Hamman, 2006). For every kilogram of weight loss, there was a 16% reduction in diabetes incidence (Hamman, 2006).

Following this evidence, the Centers for Disease Control and Prevention (CDC) created the National Diabetes Prevention Program (DPP) in 2010. Specifically, DPP includes CDC-approved curricula, a trained cadre of health coaches, and support groups of peers. These evidence-based components are informed by the DPP goals and findings from the DPPRG: 5% weight loss, at least 150 min of physical activity per week, and engagement in sessions with coaches and peers in the 12-month program (Albright and Gregg, 2013). However, to increase the coverage of the DPP to high-risk populations, the program standards were amended to allow for telehealth and hybrid (in-person/remote) program implementation in 2018.

While a wealth of literature suggests the DPP and other lifestyle change in-person programs are cost-effective approaches to diabetes risk reduction (Gruss, 2019, Albright and Gregg, 2013), less is known about the effectiveness of remote delivery of the DPP (Vadheim, 2017), specifically looking at weight loss as a key outcome. The literature that does exist on remote/distance weight loss interventions shows promising results (Tate et al., 2003, Ackermann, 2014, Ma, 2013, Kanaya, 2012, Sepah et al., 2014). This study sought to contribute to the body of evidence on the effectiveness of remote DPP programs on weight loss. The objective of this study was to estimate the associations between completion of the Brook+ 52-week digital DPP program and weight loss. We hypothesized that Brook+ program completion would result in clinically meaningful weight loss.

## 2 Methods

### 2.1 Study sample

Our study sample was drawn from a population of members of a single health insurance company in Western New York that registered to use Brook+ between December 2020 and December 2022. Brook+ is a commercially available digital DPP program offered and operated by Brook, Inc. The 52-week remote program delivers CDC curricula via a mobile app, instructional webinars and videos, personal health coaching, and group support in addition to remote monitoring devices (wireless scale and fitness tracker) to enrolled members. The insurer provided Brook with a list of potentially eligible members based on plan type, medical history, and claims data. Brook conducted outreach via mail or email to these individuals to notify them about the program and invited them to complete the CDC Prediabetes Risk Test (https://services.brook.health/enrollment/#/questions)^1^ (CDC, 2021).

Individuals were eligible for Brook+ if they were at least 18 years old, not previously diagnosed with diabetes, were not pregnant, had a body mass index (BMI) greater than or equal to 25 kg/m^2^ (23 kg/m^2^ for individuals of Asian race) with health insurance that covers remote DPP services. Eligible members joined through the Brook+ website or smart phone application, completed an intake survey^1^ (demographic information, weight, height, and information on weight loss motivations and experiences) and verified their health insurance information. After registering online, users accessed Brook+ via the smartphone application or on a computer. Withings© Body scales were sent to users’ homes following registration. Then, after 4 weeks of active participation in the program, users received a voucher for a free Fitbit© fitness tracking device.

Between December 2020 and December 2022, Brook+ enrolled 12,462 users. After removing 179 individuals without health insurance and 1,177 without starting weight or in the program less than 3 months, we included a descriptive sample of 11,106 Brook+ users. Moreover, after removal of 5,590 users with no follow-up weights collected, our analytic sample included 5,516 Brook+ users.

### 2.2 Data collection

Brook data collection began after the health insurance status of an eligible and enrolled member was verified by Brook personnel. Brook devices required no syncing and transmission of data occurred longitudinally via cellular signal from the devices. Weights were transmitted directly if scales were linked to the Brook+ application. Physical activity was recorded automatically by the Fitbit. Weights, physical activity minutes, and meals consumed were also manually input by users. Brook+ data were collected in such a way that, if a user engaged in any way (lessons, coach interactions, weighing, etc.), they will have a day of observation. Thus, we created distinct descriptive and analytic samples to capture the engagement of users.

### 2.3 Measures

The primary outcome of interest in our study was weight loss operationalized in two ways: 1) whether the individual lost 5% of their weight at enrollment by their last weight measurement (DPRP outcome standard) and 2) the difference between starting weight and most recent recorded weight (in pounds). We defined program completion as at least 48 weeks of program participation. We also distinguished between program status of individuals – those who disenrolled from the program before 48 weeks, those currently active, and those who completed the program. Other study variables included demographics (gender, age), health insurance type (private, Medicare, Medicaid) as a categorical variable, self-reported physical activity level at baseline (sedentary, light, or moderate/heavy exercise), body mass index (BMI; kg/m^3^) at baseline (recorded at intake, calculated from baseline weight and height values). Measures of engagement with program components included any interaction with a health coach (yes/no), total minutes of recorded physical activity per week, meals logged, and whether a user engaged in all three.

### 2.4 Statistical analysis

We presented frequencies with percentages and medians with 25th and 75th percentiles for categorical and continuous demographic and baseline characteristics and engagement measures, respectively, by program status. Additionally, we compared user demographic and baseline characteristics based on inclusion in the descriptive sample. Our main analysis explored the association between Brook+ program completion and 5% weight loss using generalized linear models with binomial family and logit link specifications to estimate odds ratios (OR) and 95% confidence intervals (95% CI) adjusted for age (years) and gender. We stratified our main analysis model and included interaction terms in the generalized linear models to assess potential effect measure modification by health insurance type. To assess associations between program engagement components and weight loss, we used separate linear regression models adjusted for age and gender to estimate weight loss (in pounds) with high levels of engagement (greater than the median) for health coach interactions, number of recorded physical activity minutes, number of meals logged, or all three over the course of a user’s program participation. Given that users can manually enter weights, we applied single imputation to recode implausible values (<100 lb/45.4 kg) to the median weight. Missing values of health insurance type (2%) were imputed to the most common category (private health insurance) for analysis. Covariate selection was informed by *a priori* knowledge of digital health programs and weight. We did not adjust for starting weight or BMI as our outcomes were calculated based on starting weight. All statistical analyses were conducted using Stata version 17 (StataCorp, , 2021).

### 2.5 Ethics statement

All costs of participation in the Brook+ program were covered by the users’ health insurance carrier. This study was approved by the University at Buffalo Institutional Review Board (IRB 00006667) and informed consent was waived. Study information was reported based on the Strengthening the Reporting of Observations Studies in Epidemiology (STROBE) guidelines.

## 3 Results

**Fig. 1:**
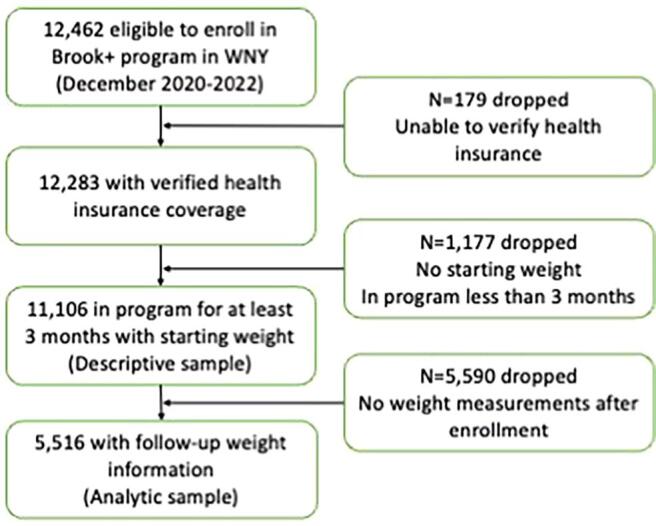
Flow of inclusion of Brook+ users into the study sample.

Fig. 1 presents the flow of participants into the study based on inclusion criteria. The comparisons presented in Supplemental Table 1 indicate that excluded users were similar in age and gender, but were more likely to be missing information on baseline physical activity level and health insurance than those included in the descriptive sample. The final descriptive sample for this study included 11,106 Brook+ users enrolled between December 2020 and December 2022 with a starting weight value at baseline and at least 3 months in the program. Twenty-two percent of Brook+ users achieved 5% weight loss. Table 1 presents descriptive statistics of the sample overall and by program completion status. Overall, the median (25th, 75th percentile) age of users was 61 years (51, 70), most (73%) were female with private health insurance (60%) and a median BMI of 32 (28, 36) kg/m^2^ (Table 1). Most of those who enrolled (76%) completed the 52-week Brook+ program. Users who completed the program were older (median age = 62 years), were more likely to engage in moderate/hard physical activity at baseline, and more likely to be enrolled in Medicare. Most (81%) interacted with a health coach while 35% logged any meals. Moreover, recorded physical activity was low across all program status categories (median = 0 minutes).

**Table 1:** Demographics, baseline characteristics, and measures of engagement among Brook + users in Western New York, by program completion status; 2020–2022.

	N (%) or Median (25th, 75th)			
	Overall	Did not complete program	Active	Completed program
Number of patients	11,106 (100)	1,479 (13)	1,221 (11)	8,406 (76)
Age (years)	61 (51, 70)	60 (47, 69)	54 (42, 64)	62 (52, 70)
Sex				
Female	8,086 (73)	1,113 (75)	877 (72)	6,096 (73)
Male	3,020 (27)	366 (25)	344 (28)	2,310 (27)
*Baseline characteristics*				
BMI (kg/m^2^)	32 (28, 36)	32 (28, 37)	32 (28, 36)	32 (28, 36)
Physical activity level				
Sedentary	3,250 (29)	475 (32)	395 (32)	2,380 (28)
Light	5,002 (45)	642 (43)	549 (45)	3,811 (45)
Moderate/Hard	2,826 (25)	357 (24)	277 (23)	2,192 (26)
Missing	28 (0.25)	5 (0.34)	0	23 (0.27)
Health insurance type				
Private	6,599 (60)	865 (58)	657 (54)	5,077 (60)
Medicare	3,705 (33)	337 (23)	253 (21)	3,115 (37)
Medicaid	583 (5)	80 (5)	310 (25)	193 (2)
Missing	219 (2)	197 (13)	1 (0.1)	21 (0.3)
*Measures of engagement*				
Ever interacted with a health coach				
No	993 (9)	129 (9)	101 (8)	763 (9)
Yes	8,953 (81)	1,136 (77)	985 (81)	6,832 (81)
Missing	1,160 (10)	214 (14)	135 (11)	811 (10)
Recorded any meals				
No	5,972 (54)	827 (56)	669 (55)	4,476 (53)
Yes	3,974 (35)	438 (29)	417 (34)	3,119 (37)
Missing	1,160 (10)	214 (15)	135 (11)	811 (10)
Total weekly recorded physical activity (minutes)	0 (0, 85)	0 (0, 53)	0 (0, 97)	0 (0, 88)

BMI: Body mass index.

Table 2 presents the results of our main analysis exploring the adjusted associations between program completion and at least 5% weight loss for the pooled sample and stratified by health insurance type. Our analytic sample was comprised of 5,516 users with follow-up weight information. There were 557,166 weights with an average (SD) of 98 (80) weights per user. In the pooled sample, those who completed the Brook+ program had statistically significant increased odds of 5% weight loss (OR = 1.21 95 % CI: (1.02 – 1.44; Table 2). Those with private health insurance had 21% higher odds of 5% weight loss with program completion compared to those who did not (OR = 1.21 95 % CI: (0.98 – 1.50). Among the Medicare and Medicaid populations, those who completed the program had 33 and 13% higher odds of 5% weight loss, respectively, compared to those who did not complete the program. These differences in odds of weight loss with program completion between health insurance types were not significant (p-interaction = 0.791; Table 2).

**Table 2:** Adjusted^1^ odds ratios and 95% confidence intervals estimating the association between Brook+ program completion and 5% weight loss in Western New York; 2020–2022.

	Pooled	Private	Medicare	Medicaid
Completed program (ref = No)	**1.21**	1.21	1.33	1.13
	**(1.02–1.44)**	(0.98 – 1.50)	(0.95–1.87)	(0.62 – 2.06)
Age (years)	**1.02**	**1.02**	1.01	1.02
	**(1.01–1.02)**	**(1.01–1.03)**	(0.99–1.02)	(0.99–1.04)
Female (ref = Male)	1.14	1.05	**1.37**	0.74
	(0.98–1.33)	(0.87–1.28)	**(1.05–1.79)**	(0.37 – 1.50)
*p-interaction*		0.791		
*Number of users*	5,516	3,574	1,648	294

Associations are estimated using generalized linear models with a binomial family and logit link specification.

Boldface indicates statistical significance.

1 Models are adjusted for user age and gender.

The results of the adjusted linear regression models that estimated pounds of weight loss achieved with engagement in the program are presented in Table 3. On average, high coach interaction (median = 2), physical activity (median = 1,425 min), and meal logging (median = 32) throughout participation in the program were all associated with more than three pounds (1.36 kg) of weight loss (p < 0.001; Table 3). Users who were highly engaged (those who reported high engagement with all three measures) lost more than four pounds (1.81 kg), on average (p < 0.001).

**Table 3:** Adjusted^1^ coefficients of weight loss with high Brook + program engagement in Western New York; 2020–2022 (N = 5,516).

	Weight loss (pounds)	SE	p-value
Coach interactions	3.42	0.29	**<0.001**
Physical activity	3.21	0.28	**<0.001**
Meals logged	3.43	0.29	**<0.001**
Highly engaged with all three measures	4.11	0.31	**<0.001**

1 Models are adjusted for user age and gender. Boldface indicates statistical significance.

## 4 Discussion

In this study, we examined weight loss associated with participation in a digital DPP across three insured populations: private, Medicare, and Medicaid. This study of individuals at-risk for diabetes participating in the Brook+ program that includes coaching, education, and objective monitoring of weight and activity revealed that program retention and engagement were high. Our main analyses found significant positive associations between program completion and 5% weight loss overall. In the subpopulations by health insurance type, odds of weight loss were highest among those enrolled in Medicare, though statistical significance was lost. High engagement with the program via coach interactions, physical activity, and meal logging were all significant predictors of weight loss.

Despite the observational nature of our study design, our findings are consistent with other studies, including randomized controlled trials. Brook+ users in this study lost an average of 4% body weight, which is comparable to the average percent weight loss observed in other populations (Ali et al., 2012, Joiner et al., 2017). While Ali and colleagues conducted a *meta*-analysis of weight loss in programs with various modes of DPP delivery (Ali et al., 2012), both our study findings and those of Joiner and colleagues which look at remote DPPs agree with 4% average weight loss (Joiner et al., 2017), suggesting remote DPP may have comparable effectiveness to in-person delivery modes. Tate and colleagues found an internet-based weight loss program with e-counseling reduced average weight by 4.1 kg and 45% of participants reached 5% weight loss by six months (Tate et al., 2001) and by 4.4 kg and 4.8% of their body weight after 12 months (Tate et al., 2003). Similarly, Vadheim and colleagues found that a telehealth translation of the DPP provided to rural communities resulted in 56% of the sample losing at least 5% body weight (Vadheim, 2017). It is also worth noting that the Vadheim study found no differences in weight loss or program engagement outcomes across telehealth or on-site DPP groups, suggesting digital delivery may be just as effective as on-site programs.

Average weight loss in these studies was similar to that observed in our study (9 lb/4 kg weight loss) and 22% of our sample lost at least 5% starting body weight. And, we applied the same 12-month duration of exposure, which most other studies used to test program effectiveness, to optimize comparability to other studies. Comparable to the weight loss findings with Brook+ program engagement, participants in the *Live Well, Be Well* study that included a telephone-based diet and physical activity education and skills building component was shown to promote weight loss, with average weight loss of 2 lb achieved at 6 months of program participation (Kanaya, 2012).

Our results suggest that high engagement with program components is a significant predictor of weight loss in the Brook+ program. Similarly, Sepah and colleagues found that completing logins and group participation were significant predictors of weight loss, though coach conversations were not significantly associated with weight loss (Sepah, 2017). A secondary analysis of 273 individuals enrolled in a 4-month digital DPP found that participants increased exercise frequency by 1.7 days per week and lost 13 lb, on average (Alwashmi, 2019), suggesting that even in the short-term, lifestyle changes can have great health benefits for individuals with prediabetes. Additionally, an analysis of 2,037 participants in the Livongo DPP found that highly engaged individuals lost an average of 6.6% of starting body weight (compared to 5.1% among the overall sample) and that food logging, lesson completion, and physical activity were all positively associated with weight loss (Stefanie, 2020). As in our study, food logging was shown to have the largest magnitude association with weight loss. Moreover, coach interactions were drivers of other measures of engagement, indicating that coaches are an important point of contact for user needs and engagement in digital DPPs. Similarly, in Brook+, coaches review engagement parameters and guide users accordingly to ensure optimal program benefit.

Our study findings of increased, though null, odds of 5% weight loss in the subsamples of health insurance types agree with those of significantly greater weight loss among the 65 years and older population receiving a digital DPP compared to a standard care group (Katula, 2022). Moreover, Gruss et. al. found increased odds of achieving 5% weight loss among the combined Medicare/Medicaid sample when compared to grant funding as a payment source (Gruss, 2019).

### 4.1 Strengths

This study was conducted among a sample of individuals from a single payer at high-risk for diabetes who were enrolled in a digital commercial asynchronous DPP. Users were provided with cellular instrumentation (weighing scales and fitness trackers) linked to a Brook+ smartphone or web-based application which would automatically transmit objective measures of weight and engagement to a secure server. We have a relatively large sample size from various health insurance types which may serve to represent different demographic backgrounds of users. We assessed program completion and weight loss outcomes using definitions that enable comparison to the extant literature and are in-line with DPPRG goals (5% weight loss).

### 4.2 Limitations

Loss to follow-up in this study may produce selection of users into this study in such a way that introduces bias. While much of the data are collected automatically through cellular devices, users can input their own values and information, thus introducing potential misclassifications. We appreciate the findings from our main analyses may be due to selection biases as we note those who were excluded were largely enrolled in Medicaid and the included sample was older, potentially indicating less understanding of the technology needed for the Brook+ program. This, in turn, may have altered engagement patterns including recorded weights, of which more than 50% were missing. We also lack detailed information on the underlying demographic information of Brook+ users as Brook data systems were not designed with research in mind. Thus, we are also limited in the data that are collected and available for study, such as demographics and other potential confounders including medication use, healthcare utilization, enrollment in other diabetes prevention or weight loss programs, so confounding is likely to bias our findings. Given the variability of weight loss drug prescribing practices, differential coverage by health insurance type, and that all users have health insurance, the potential biases introduced by confounding by medication use are unknown and should be explored in future research. Moreover, there is no systematic data collected related to reasons for program discontinuation for those who did not complete the 52 weeks, which potentially introduces a selection bias whereby the healthiest individuals are more likely to have program success. However, review of the open-ended free text responses provided by certain users when they requested program discontinuation more so indicates dissatisfaction with the program or time constraints as the principal reason, minimizing our concerns of confounding by health status.

Generalizability of these study findings are limited as our sample was comprised of willing individuals enrolled in a single health insurance program in Western New York that would need some degree of technological literacy to engage with the program. Brook receives only limited information of patients deemed eligible for the DPP by their health insurer. Consequently, we are limited in our comparisons of our study sample characterstics to those who were not enrolled. Furthermore, the initial sample of users enrolled into Brook+ during the COVID-19 pandemic, those included in this study sample, may likely differ from the target population of those with prediabetes in a way that could bias our results.

## 5 Conclusions

Given that weight loss has been shown to be the most important predictor of diabetes risk reduction, weight reduction interventions represent critical public health strategies. Particularly, remote DPP modalities present cost-effective and flexible approaches to lifestyle interventions. More research is needed to evaluate whether these programs reach high-risk populations and to examine longer-term outcomes.

## CRediT authorship contribution statement

**Sarah LaPointe:** Conceptualization, Data curation, Formal analysis, Methodology, Writing – original draft, Writing – review & editing. **Michael Merrill:** Conceptualization, Supervision, Writing – review & editing.

## Declaration of Competing Interest

The authors declare the following financial interests/personal relationships which may be considered as potential competing interests: Sarah LaPointe has been employed as a part-time Researcher at Brook, Inc. since May 2022. Michael Merrill is Chief Medical Officer at Brook, Inc. Brook, Inc. provided funding and financial support for this study and publication.

## Data Availability

The data that has been used is confidential.

## Footnotes

1 The information collected in these prescreening surveys was not systematically collected during the study period.
