# Supplementary material for: Weight loss and lifestyle change among high-risk individuals enrolled in a digital diabetes prevention program: A longitudinal study of private and public health insurance members in Western New York
Source: Prev Med Rep. 2023 Nov 10;36:102507. doi: 10.1016/j.pmedr.2023.102507 (PMC10728453; doi:10.1016/j.pmedr.2023.102507)
Supplement: Supplementary data 1 [file mmc1.docx]

**Supplemental Table 1. Comparison of characteristics between included and excluded Brook+ users in Western New York; 2020-2022.**

| Mean or % | |  |  |
| --- | --- | --- | --- |
| User characteristics | Included | Excluded^1^ | p-value |
| Age (years) | 59.5 | 58.9 | 0.487 |
| % Female | 72.8 | 73.6 | 0.553 |
| Starting physical activity level (%) |  |  | **<0.001** |
| Sedentary | 29.3 | 27.1 |  |
| Light | 45.0 | 38.5 |  |
| Moderate/Hard | 25.5 | 21.2 |  |
| Missing | 0.25 | 13.2 |  |
| Health insurance type (%) |  |  | **<0.001** |
| Private | 59.4 | 16.1 |  |
| Medicare | 33.4 | 13.2 |  |
| Medicaid | 5.3 | 2.7 |  |
| Missing | 1.9 | 68.1 |  |
| 11,106 | 1,356 |  |  |

^1^ Missing information on starting weight and in the program for less than three months.

BMI: Body mass index; SD: Standard deviation. Boldface indicates statistical significance.
